# Treatment of an Osteochondral Defect of the Trochlea Using Matrix-Induced Autologous Chondrocyte Implantation and Proximal Tibia Autologous Bone Grafting

**DOI:** 10.1016/j.eats.2025.103507

**Published:** 2025-03-15

**Authors:** Rishi Trikha, Thomas J. Kremen, Paul J. Walker, Kristofer J. Jones

**Affiliations:** Department of Orthopaedic Surgery, University of California, Los Angeles, Los Angeles, California, U.S.A.

## Abstract

Chondral and osteochondral defects of the knee are painful and disabling in the short term, can lead to accelerated osteoarthritis in the long term, and have relatively poor healing potential. Although many cartilage restoration procedures exist for focal cartilage defects, matrix-induced autologous chondrocyte implantation (MACI) for the appropriate patient has evolved as a reliable technique with encouraging long-term outcomes. Despite the reported favorable outcomes, there remains a relative dearth of literature on the use of MACI with autologous bone graft. This article presents a modified MACI sandwich technique that uses only 1 scaffold in combination with autologous bone graft from the proximal tibia to address an osteochondritis dissecans lesion of the lateral trochlea. This technique allows for both a stable MACI single-layer scaffold and a relatively efficient operative workflow.

Symptomatic chondral and osteochondral defects of the knee are becoming increasingly common, with up to 66% of patients undergoing knee arthroscopy having articular cartilage pathology and 11% showing cartilage defects that are suitable for a cartilage repair procedure.^1^^,^^2^ Restorative surgical techniques of these defects have continued to evolve over the past few decades, with microfracture, osteochondral autograft transfer, osteochondral allograft transplantation, and autologous chondrocyte implantation (ACI) becoming the mainstays in treatment.

The ACI technique, in particular, has seen advances over the past few decades, with third-generation techniques overcoming the original challenges associated with periosteum-covered patches. The matrix-induced autologous chondrocyte implantation (MACI) technique uses cultured autologous chondrocytes placed directly onto the surface of a porcine type I/III collagen membrane (Vericel, Cambridge, MA), eliminating the need for a periosteal patch. Furthermore, MACI has shown high levels of patient satisfaction as well as radiographic graft survivorship more than 10 years after index surgery for focal chondral lesions.^3^ Although generally positive results of MACI procedures for chondral defects continue to be observed, there is a relative dearth of literature on the treatment of osteochondral defects with autologous bone graft. One such study showed superior outcomes for a traditional MACI “sandwich” technique when compared with autologous bone grafting alone when treating a deep osteochondral lesion in the knee.^4^

This article presents a modified MACI sandwich technique that uses only 1 scaffold in combination with autologous bone graft from the proximal tibia to address an osteochondritis dissecans (OCD) lesion of the lateral trochlea (Fig 1). The second stage of the MACI procedure is described in this article. The first stage involves arthroscopic chondrocyte harvest from the intercondylar notch using a large ring curette (Fig 2). These chondrocytes are then grown in vitro and seeded onto a membrane to create the MACI scaffold to be used in the second stage. The surgical indications and contraindications of this technique are summarized in Table 1.

**Fig 1:**
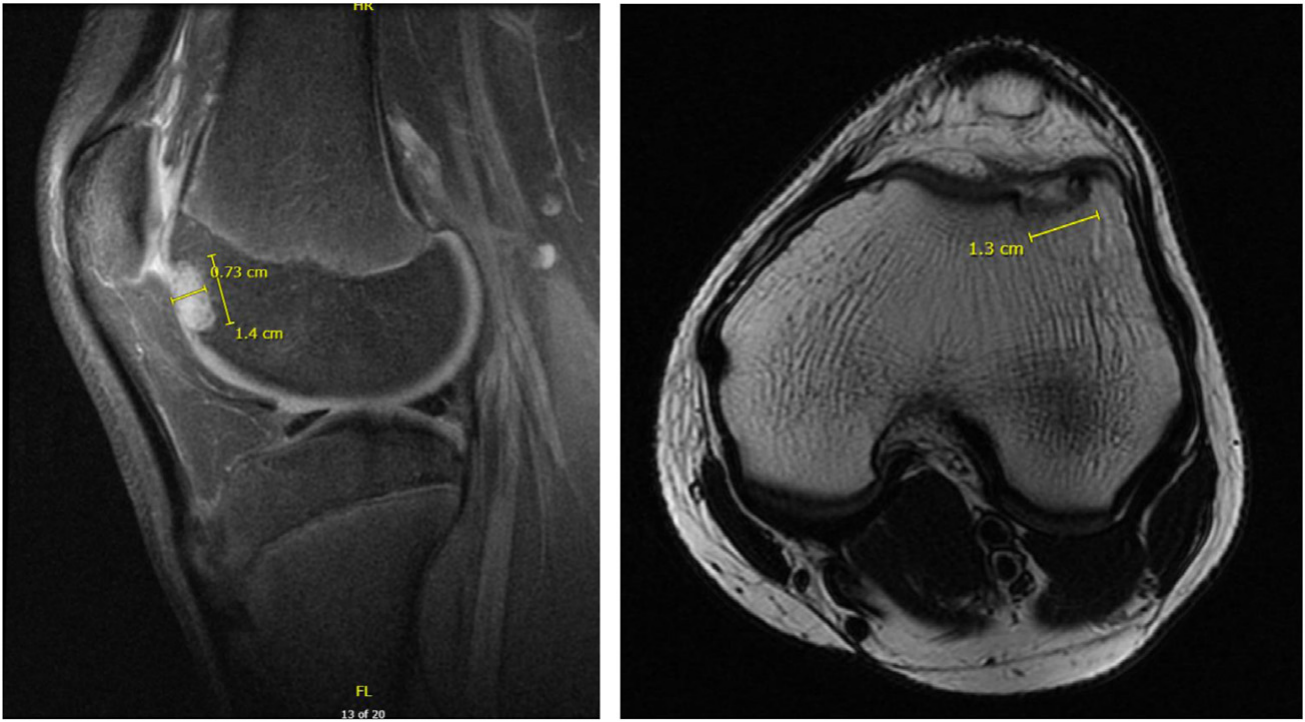
Magnetic resonance imaging scans of left knee in 16-year-old male patient showing lateral trochlea osteochondral defect measuring roughly 14 mm × 13 mm and involving subchondral bone.

**Fig 2:**
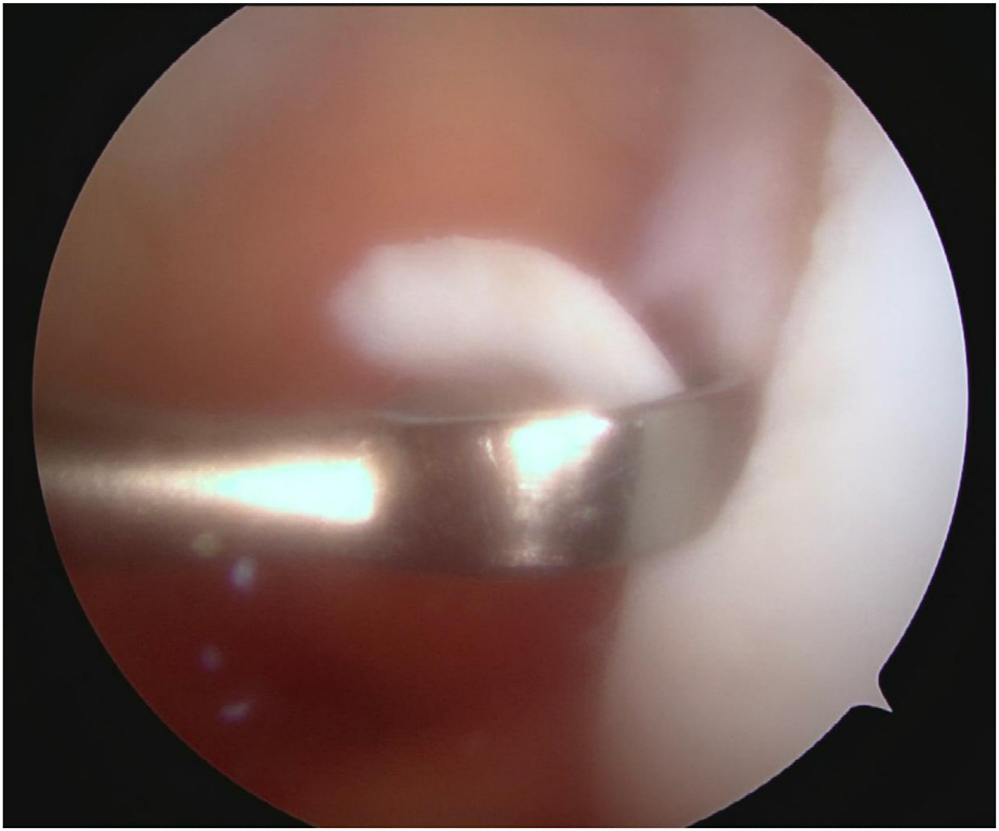
The patient is placed supine and, after standard sterile preparation and draping, the left lower extremity is exsanguinated and the tourniquet is inflated to 250 mm Hg. A standard anterolateral viewing portal is established and a diagnostic examination ensues. An anteromedial portal was then established under direct visualization. Chondrocytes were then harvested from the intercondylar notch using the large ring curette during the first stage of the procedure.

**Table 1:** Indications and Contraindications for MACI Transplantation for Osteochondral Defects

Indications
Young patients who are not candidates for arthroplasty
Body mass index < 35
Femoral condylar, trochlear, patellar, or tibial full-thickness, symptomatic osteochondral lesions measuring ≥2 cm^2^
Cavitary defect in bone
Contraindications
Inflammatory arthritis
Active or recent infection
Lower-extremity malalignment
Ligamentous instability
Multi-compartmental osteoarthritis

MACI, matrix-induced autologous chondrocyte implantation.

## Surgical Technique

**Fig 3:**
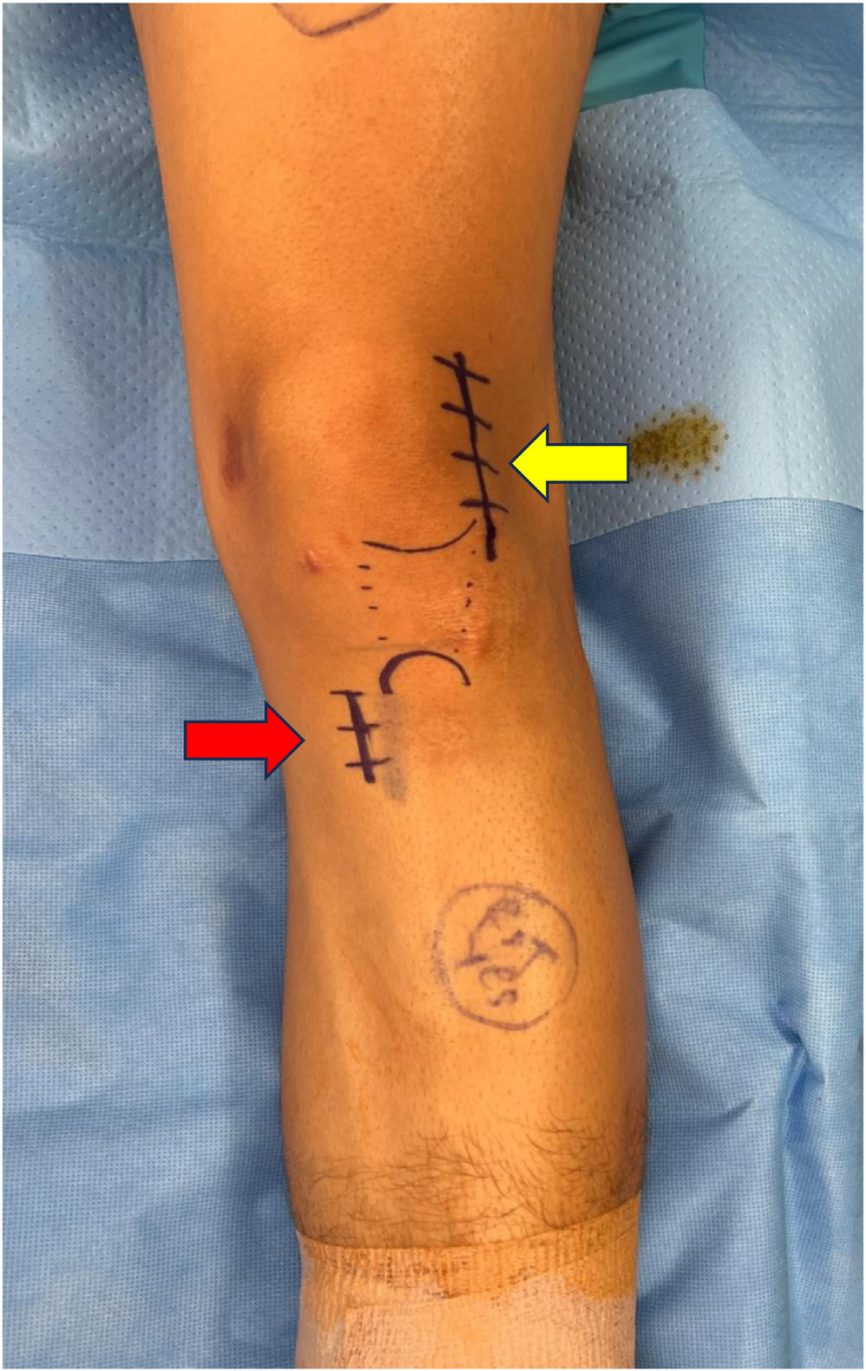
The planned incisions are marked. For the proximal tibia bone graft harvest, a roughly 3-cm incision centered above the pes anserinus and just medial and inferior to the tibial tubercle (red arrow) is planned. To access the lateral trochlea, a roughly 8-cm incision centered over the lateral-most aspect of the patella (yellow arrow) is planned.

This surgical technique is described in Video 1, as well as Table 2. The patient is placed supine on the table, and after general anesthetic induction, an evaluation of the operative knee is performed with the patient under anesthesia. After standard sterile preparation and draping, the operative extremity is exsanguinated and the tourniquet is inflated to 250 mm Hg. The planned incisions, described later, are then marked (Fig 3). A diagnostic arthroscopic examination ensues, and the contained OCD lesion of the lateral trochlea is inspected. Attention is then paid to the autologous bone graft harvest from the proximal tibia.

**Table 2:** Step-by-Step Guide to Performing Technique

Step	Description
1	Perform diagnostic arthroscopic examination using standard portals to localize and characterize the osteochondral defect.
2	Turn attention to open proximal tibia autologous bone harvest. Make an incision just above the pes anserinus, and dissect down to bone.
3	Use the 10-mm OsteoAuger to ream and harvest proximal tibia bone graft, and fill the void created with cancellous bone allograft chips.
4	Turn attention to open treatment of the OCD lesion. Make another incision centered over the lateral-most aspect of the patella, and complete lateral arthrotomy.
5	Identify the osteochondral defect, and choose the appropriate template size to fully, circumferentially encapsulate the defect.
6	Drill a 2.4-mm Kirschner wire through the center of the sizing template, and proceed to ream to a depth of 5 mm. If there is a deeper area of abnormal bone, ream to a depth that allows for a shallow base with bleeding, healthy-appearing cancellous bone.
7	Turn attention to the MACI scaffold. Cut the scaffold to measure the previously measured template.
8	Turn attention back to the osteochondral defect. Pack with autologous bone graft and secure with fibrin glue.
9	Place the MACI scaffold, with the cell-layer side toward the bone over the bone graft, and again secure with fibrin glue. Note that there is an option at this point to further secure the MACI scaffold with No. 6-0 Vicryl suture.
10	Take the knee through a passive range-of-motion test to ensure stability of the scaffold.
11	Perform appropriate wound closure.

MACI, matrix-induced autologous chondrocyte implantation; OCD, osteochondritis dissecans.

**Fig 4:**
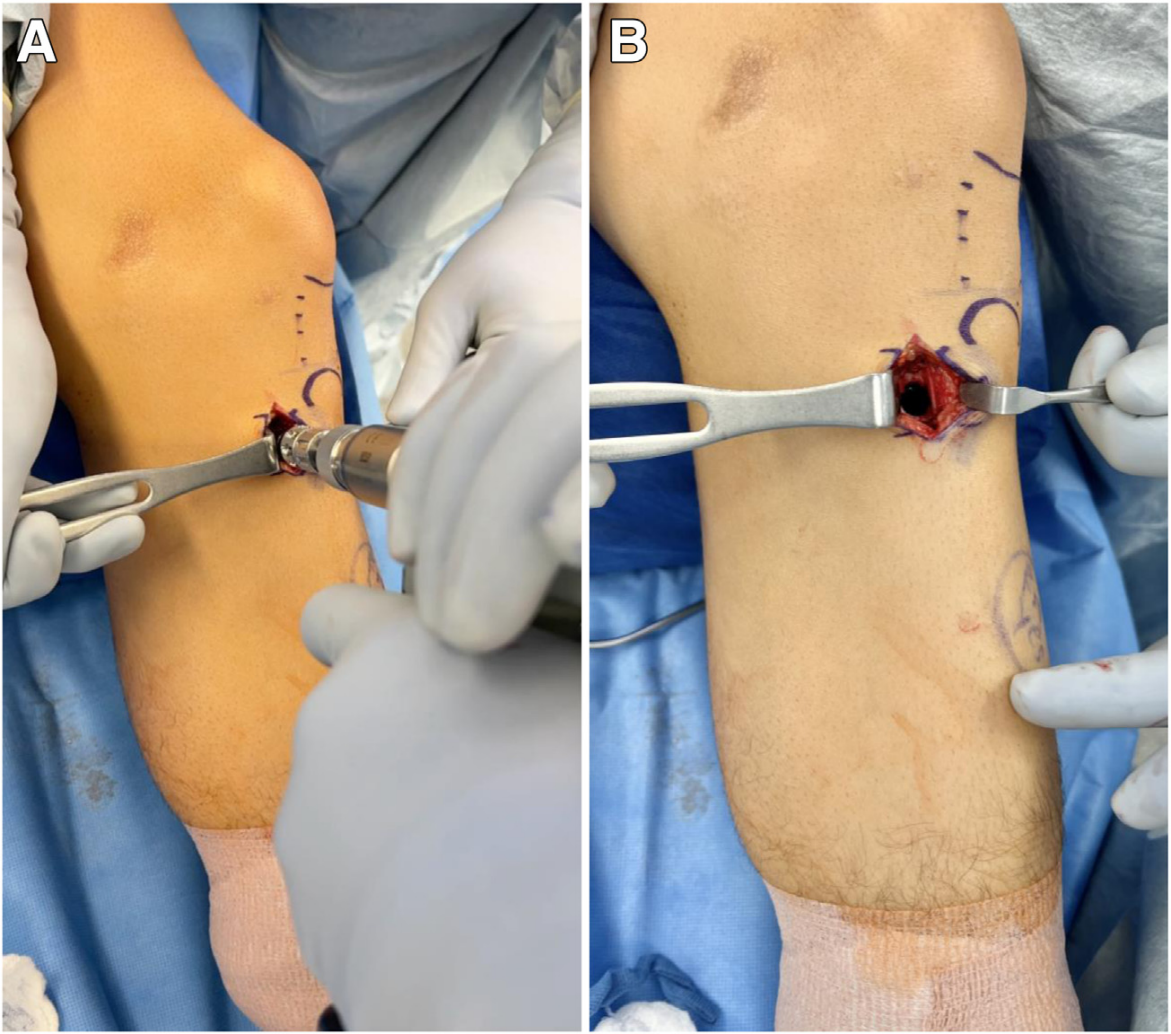
(A) The OsteoAuger is used to ream and harvest 8 to 10 cm^3^ of bone graft in the proximal tibia. This amount is standardized based on the amount of subchondral bone involvement on magnetic resonance imaging and can be augmented with allograft. (B) The resulting bone void is shown and is packed with cancellous allograft chips.

A 3-cm vertical incision centered just above the pes anserinus and just medial and inferior to the tibial tubercle is made. Dissection is carried down to tibial bone. The 10-mm OsteoAuger (Arthrex, Naples, FL) is then used to ream and harvest roughly 8 to 10 cm^3^ of bone graft from the proximal tibia (Fig 4A). This amount is standardized based on the amount of subchondral bone involvement on magnetic resonance imaging and can be augmented with allograft. Cancellous allograft chips are subsequently placed into the bone void created (Fig 4B).

**Fig 5:**
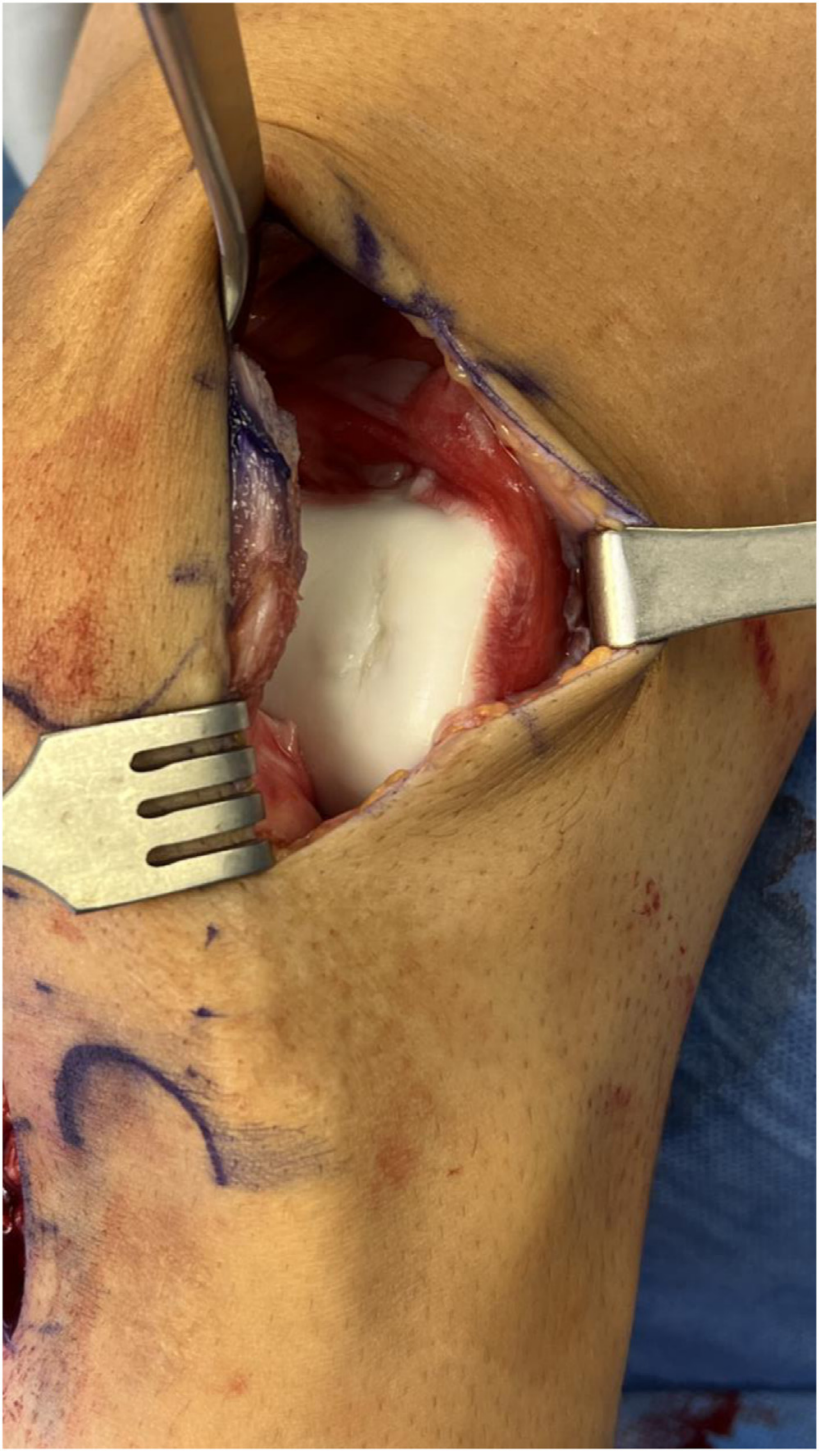
A lateral arthrotomy is made to inspect the osteochondral defect.

**Fig 6:**
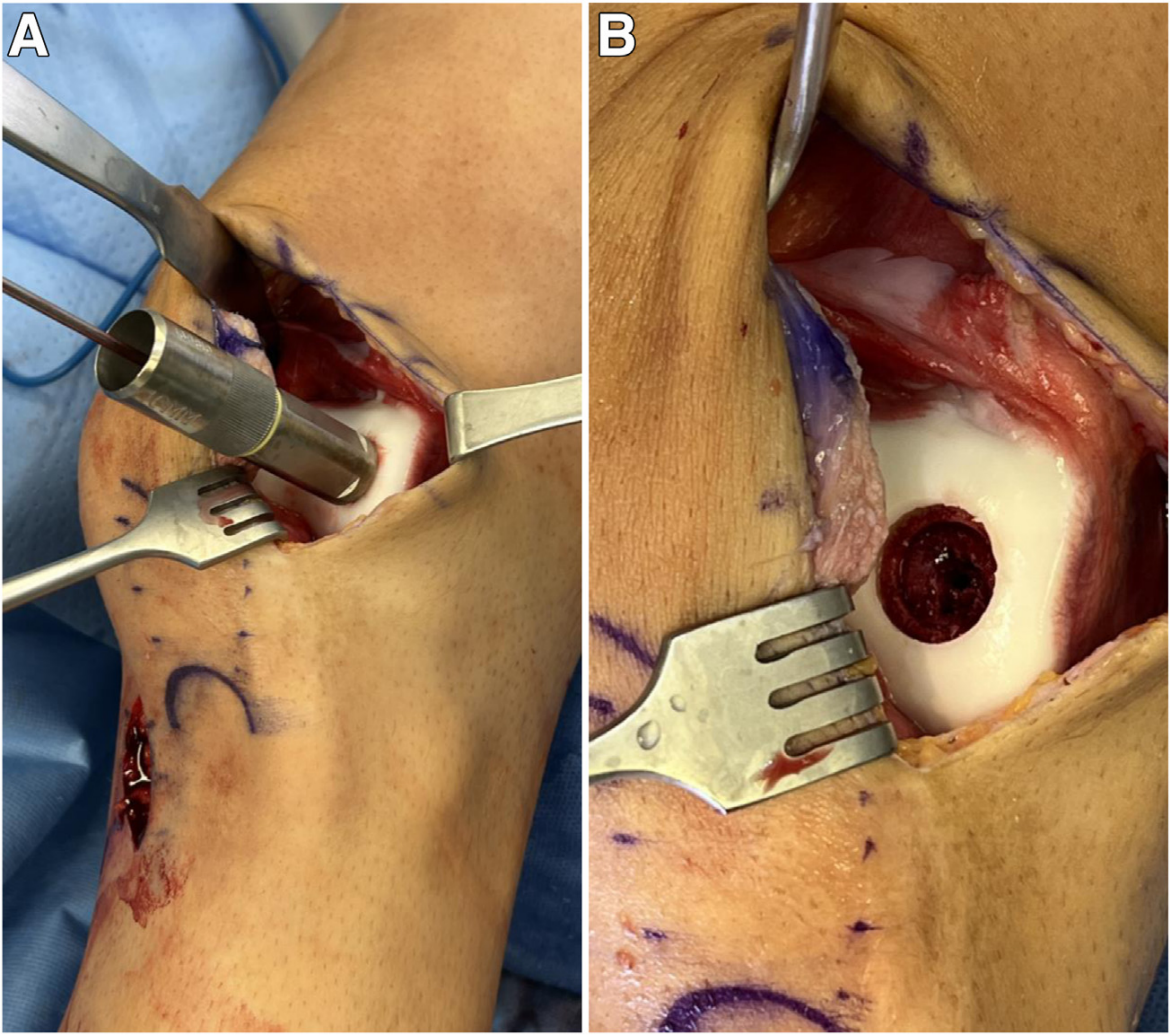
(A) The sizing template from the JRF Ortho osteochondral allograft instrumentation tray is used to measure the defect such that the entire defect is encapsulated by the circumference of the template. The appropriate template size is determined by finding the smallest size that encompasses the defect while being on the margin of what is considered normal cartilage. In this case, the 16-mm sizing template completely encompasses the defect. A 2.4-mm Kirschner wire is then placed centrally through the sizing template. (B) The sizing template is removed, and a 16-mm reamer is used to ream to a depth of 5 mm until bleeding, healthy subchondral bone is reached. In this case, there is a deeper area of abnormal sclerotic bone centrally. This is measured, and a 12-mm reamer is used to ream centrally and completely encompass the sclerotic bone to a depth of 8 mm until subchondral bone is reached. This results in a targetoid defect such that the central 12 mm is reamed slightly deeper than the surrounding area.

Attention is then turned to open treatment of the OCD lesion. An 8-cm incision centered over the lateral-most aspect of the patella is made, and a lateral arthrotomy is performed (Fig 5). Any loose cartilage from the OCD lesion is removed with a small ring curette. The sizing templates from the JRF Ortho (San Leandro, CA) osteochondral allograft instrumentation tray are used to measure the defect, which is determined to 16 mm in diameter. A 2.4-mm Kirschner wire is placed centrally through the sizing template (Fig 6A). The appropriately sized reamer is then used to ream circumferentially until bleeding, healthy subchondral bone is reached (Fig 6B).

**Fig 7:**
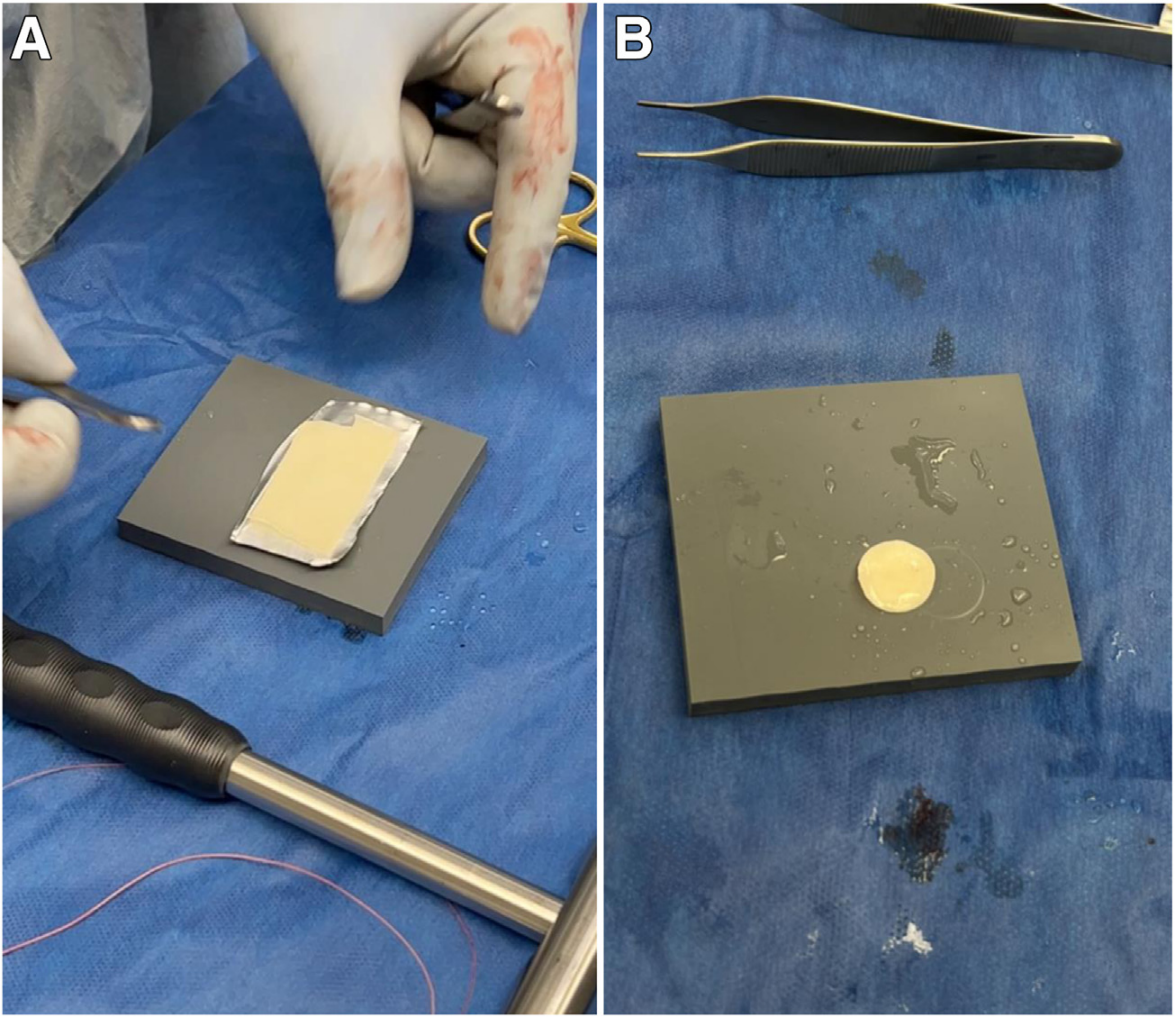
(A) The matrix-induced autologous chondrocyte implantation scaffold uses a porcine type I/III collagen membrane seeded with chondrocytes from the chondrocyte biopsy in the first stage. This scaffold is available as a single layer and has a sheet-like appearance. (B) The scaffold is laid flat and cut to the appropriate circumference from the sizing template. This is performed with the help of the JRF Ortho scoring instrument.

**Fig 8:**
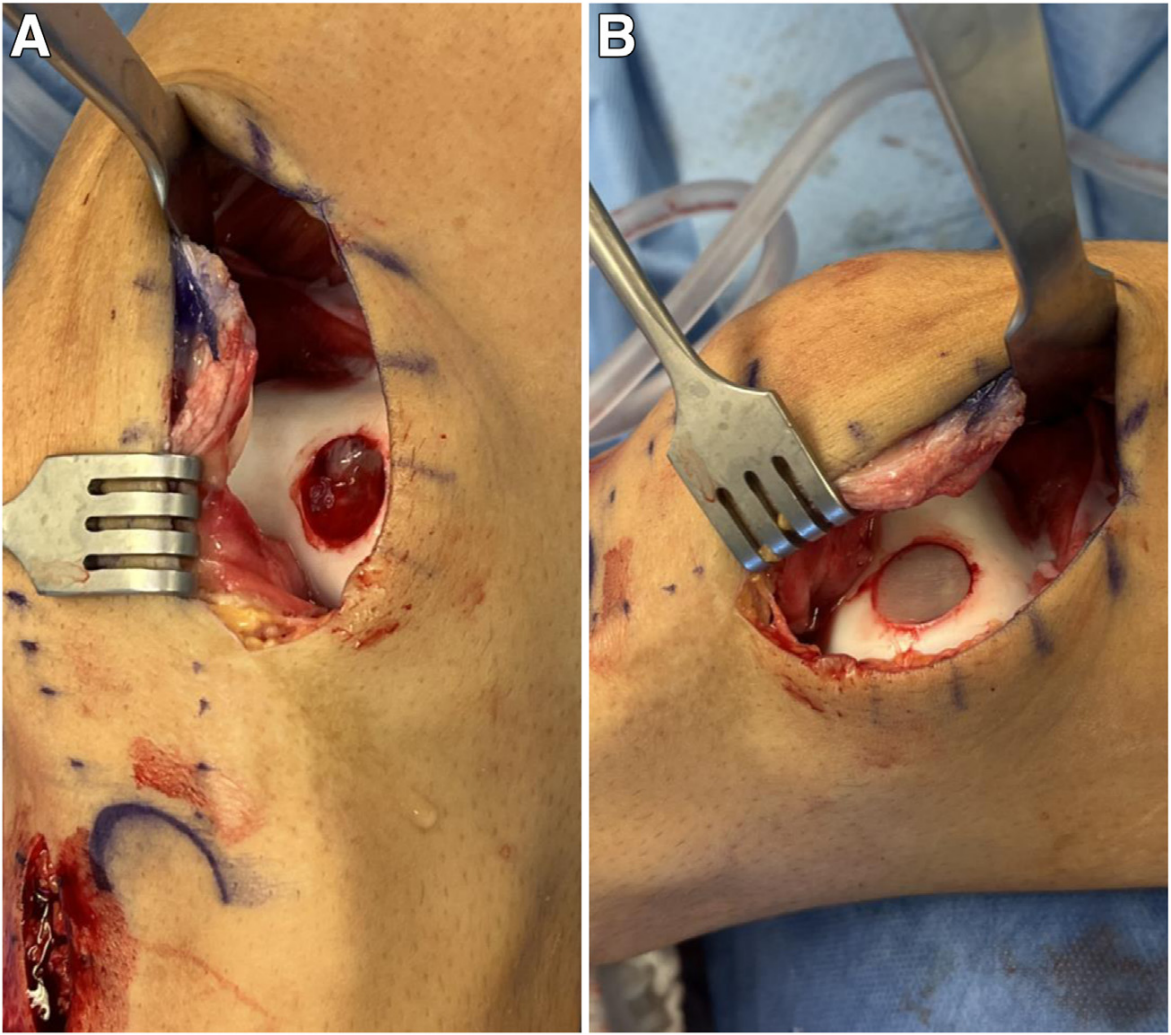
(A) Autologous bone graft from the proximal tibia harvest site is packed into the defect and secured with fibrin glue. (B) The matrix-induced autologous chondrocyte implantation scaffold is placed onto the base of the lesion with the cell side facing toward bone and is secured with fibrin glue. Light, consistent manual pressure is applied for 3 minutes and then released.

Attention next turns to the MACI scaffold, which uses a porcine type I/III collagen membrane seeded with chondrocytes and is available as a single layer with a sheet-like appearance (Fig 7A). The scaffold is laid flat and cut to the circumference of the previously measured 16 mm using the JRF Ortho scoring instrument (Fig 7B). The autologous bone graft initially harvested from the proximal tibia is then packed into the osteochondral defect so that it is even with the adjacent subchondral bone. The tourniquet is released to achieve hemostasis, and a thin layer of fibrin glue is applied to secure the bone graft within the defect (Fig 8A). The MACI scaffold is placed onto the base of the lesion with the cell side facing toward bone and is subsequently secured with another layer of fibrin glue. Light, consistent manual pressure is applied for 3 minutes and then released (Fig 8B). Four No. 6-0 Vicryl sutures (Ethicon, Somerville, NJ) are used to further secure the periphery of the MACI scaffold. The knee is taken through passive range of motion to demonstrate stability of the scaffold. The capsule of the knee is closed with No. 0 Vicryl suture. Subcutaneous No. 2-0 Vicryl sutures are then placed, followed by a No. 4-0 running Prolene stitch (Ethicon). After appropriate dressings are placed, the knee is placed in a hinged knee brace locked in extension.

The patient remains toe-touch weight bearing (approximately 20%-25%) for 4 weeks with the hinged knee brace locked in extension. Full weight bearing is allowed at 4 weeks postoperatively. The knee brace is unlocked at 6 weeks postoperatively, when the patient shows excellent quadriceps control and gait mechanics. A continuous passive motion machine is used for a total of 6 weeks postoperatively.

## Discussion

MACI has shown promising clinical outcomes and is an encouraging cartilage restorative treatment for OCD lesions in the appropriate patient.^3^^,^^5^ As ACI surgical techniques evolve, it is imperative that these techniques continue to be described in a manner that is reproducible so that long-term clinical data can be acquired. This article describes a mini-open approach to the MACI procedure using autologous bone from the proximal tibia.

There are several advantages to the described MACI technique (Table 3). This technique offers a simple method to harvest proximal tibia autologous bone. The benefits of this graft harvest include a straightforward surgical approach, anatomic proximity to the surgical site, a relatively large amount of graft, and an easy manner in which to replace the bone void created. Furthermore, the OsteoAuger system morselizes the bone, which makes it ideal for graft handling and packing. Another benefit to this surgical technique is that the need for excessive MACI scaffold handling and/or measuring is eliminated. By use of the JRF Ortho scoring instrument, the precise measurement of the diameter of the scaffold should ensure a perfect fit with the measured defect after reaming. Additionally, compared with the traditional MACI sandwich technique originally described by Bartlett et al.,^6^ which uses a 2-layer MACI scaffold, our technique uses a single-layer MACI scaffold, which potentially further limits handling and malposition of the scaffold. The use of 1 scaffold also decreases overall cost, as well as operating room time.

**Table 3:** Potential Advantages and Disadvantages

Advantages
The JRF Ortho scoring instrument allows the MACI scaffold to be cut to the exact diameter of the measured defect after it is reamed, thus eliminating the need for excessive MACI scaffold handling and placement.
When compared with the 2-layer scaffold, the single-layer scaffold in this technique further limits scaffold handling and potential malposition. The use of a single-layer scaffold also decreases overall cost and operating time.
The OsteoAuger system morselizes a relatively large amount of bone graft, making it immediately ideal for graft handling and packing.
The bone void from the proximal tibia graft harvest site can easily be replaced with allograft cancellous chips owing to great intraoperative visualization throughout the graft harvest procedure.
Disadvantages
Returning to sport typically does not occur within a year given the necessary maturation process of the MACI scaffold.
Potential pain over proximal tibia graft harvest site
Cost of MACI scaffold
2-Stage procedure

MACI, matrix-induced autologous chondrocyte implantation.

There are limitations to the described technique in addition to the expense and 2-stage nature of the procedure. One potential disadvantage of the proximal tibia graft harvest is pain over a graft site that is relatively subcutaneous. Another potential limitation is the time to integration and maturation of the MACI bone-scaffold interface postoperatively. Optimal maturation has been shown to occur roughly 1 to 2 years after implantation, which could potentially lengthen a patient’s return-to-sport time.^7^^,^^8^ It is unknown whether the stability of the bone-scaffold construct needs to be augmented with suture fixation because the fibrin glue may provide enough stability. In our opinion, additional suture fixation has little downside and is thus used. Despite these limitations, we believe that the current surgical technique provides a relatively straightforward and high-yielding bone graft harvest protocol, precise OCD defect preparation, and reliably accurate scaffold placement, all while keeping an efficient operative workflow.

## Disclosures

The authors declare the following financial interests/personal relationships which may be considered as potential competing interests: K.J.J. reports a consulting or advisory relationship with JRF Ortho and receives speaking and lecture fees from 10.13039/100007307Arthrex. All other authors (R.T., T.J.K., P.J.W.) declare that they have no known competing financial interests or personal relationships that could have appeared to influence the work reported in this paper.

## Supplementary Data

Video 1 This video demonstrates the surgical technique for the treatment of an osteochondral defect of the trochlea utilizing matrix-induced autologous chondrocyte implantation (MACI) and proximal tibia autologous bone grafting. This video will demonstrate the MACI scaffold application as well as the proximal tibia autologous bone graft harvest and application. The patient is positioned supine and the left lower extremity is exsanguinated with the tourniquet then inflated to 250 mm Hg. A diagnostic arthroscopic examination is performed utilizing a standard anterolateral portal to localize and characterize the osteochondral defect. Attention is then turned to the open proximal tibia autologous bone harvest where an incision is made just above the pes anserinus and then dissected down to bone. The 10 mm Arthrex OsteoAuger was then used to ream and harvest proximal tibia bone graft and fill the void created with cancellous bone allograft chips. The osteochondral defect is then identified utilizing an incision centered over the lateral most aspect of the patella and then completing the arthrotomy. The appropriately sized sizing template is chosen to fully, circumferentially encapsulate the defect and held in place with a 2.4 mm Kirshner wire through the center of the sizing template. The appropriately sized reamer is then used to ream to a depth of 5 mm. If there is a deeper area of abnormal sclerotic bone, ream to a depth that allows for a shallow base with bleeding, healthy cancellous bone. The MACI scaffold is then cut to the previously measured template size. Attention is then turned back to the prepared osteochondral defect and packed with autologous bone graft which is secured with fibrin glue. The MACI scaffold is placed cell-layer side towards the bone over the bone graft and again secured with fibrin glue. The knee is taken through a passive range of motion test to ensure stability of the scaffold and the wound is closed in the standard fashion.
